# Lab-on-Microsphere—FRET-Based Multiplex Sensor Platform

**DOI:** 10.3390/nano11010109

**Published:** 2021-01-05

**Authors:** Vera Kuznetsova, Viktoria Osipova, Anton Tkach, Maksim Miropoltsev, Danil Kurshanov, Anastasiia Sokolova, Sergei Cherevkov, Viktor Zakharov, Anatoly Fedorov, Alexander Baranov, Yurii Gun’ko

**Affiliations:** 1 Center of Information Optical Technology, ITMO University, 197101 Saint Petersburg, Russia; vaosipova@itmo.ru (V.O.); aptkach@itmo.ru (A.T.); miropoltsev_m@niuitmo.ru (M.M.); kurshanov_danil@itmo.ru (D.K.); avsokolova@itmo.ru (A.S.); s.cherevkov@itmo.ru (S.C.); vvzakharov@itmo.ru (V.Z.); a_v_fedorov@itmo.ru (A.F.); a_v_baranov@itmo.ru (A.B.); 2 Chemistry School, Trinity College Dublin, 2 Dublin, Ireland

**Keywords:** AgInS_2_, FRET, ternary quantum dots, cyanine dyes, microspheres, sensing, time-resolved fluorescence spectroscopy

## Abstract

Here we report on the development and investigation of a novel multiplex assay model based on polymer microspheres (PMS) encoded with ternary AIS/ZnS quantum dots (QDs). The system was prepared via layer-by-layer deposition technique. Our studies of Förster resonance energy transfer (FRET) between the QD-encoded microspheres and two different cyanine dyes have demonstrated that the QD photoluminescence (PL) quenching steadily increases with a decrease in the QD-dye distance. We have found that the sensitized dye PL intensity demonstrates a clear maximum at two double layers of polyelectrolytes between QDs and Dye molecules on the polymer microspheres. Time resolved PL measurements have shown that the PL lifetime decreases for the QDs and increases for the dyes due to FRET. The designed system makes it possible to record spectrally different bands of FRET-induced dye luminescence with different decay times and thereby allows for the multiplexing by wavelength and photoluminescence lifetimes of the dyes. We believe that PMS encoded with AIS/ZnS QDs have great potential for the development of new highly selective and sensitive sensor systems for multiplex analysis to detect cell lysates and body fluids’ representative biomarkers.

## 1. Introduction

Quantum dots (QDs) are semiconductor nanoparticles in which the motion of electrons is limited in all three spatial dimensions. QDs are well-known for tuneable photoluminescence (PL) bands [1,2,3] broad absorption spectra [1,4], great photostability in comparison to the organic dyes [5] and high PL quantum yields [6]. These unique characteristics have made them particularly interesting for both fundamental research and commercial applications [7,8]. QDs have already been applied in the fields of photonics and biomedicine, including systems for energy conversion, light-emitting devices, bioimaging tools, sensing platforms, and, since recently, assays for the multiplex analysis [9]. Optoelectronic and spectroscopic properties of the QDs strongly depend on their chemical composition, size, shape and surface chemistry [10,11], which can be controlled by implementing diverse synthetic strategies and modification techniques [12,13,14].

Quantum dots are generally synthesized of semiconductor binary compounds such as II–IV and IV–VI elements [15]. The most well-known examples include Cd- and Pb-based QDs. These QDs have been extensively investigated in the last few decades and remain prospective materials due to the well-studied chemical and optical properties and simple manufacturing techniques [16,17] However, cadmium and lead toxicity hinders further application of these quantum dots in biomedicine and, considering the latest environmental regulations, in optoelectronic technologies [18,19] The non-toxic ternary QDs, such as AgInS_2_ (AIS) or CuInS_2_ (CIS) are an excellent alternative to the traditional QDs due to the absence of toxic heavy metals in their structure [20].

Quantum dots of ternary compounds, AIS or CIS, differ from the binary QDs in optical and electronic properties. Absorption bands of ternary QDs are much broader and lack pronounced excitonic peaks [21]. Luminescence band full width at half maximum and Stokes shifts in many cases exceed hundreds of nanometres [22]. Such QDs are also known for hundred-nanosecond-long photoluminescence lifetimes that depend on the particle size and composition [21,23]. Luminescence decay kinetics is most often described as bi- or triple-exponential [24,25]. The slowest component is usually attributed to the deep donor−acceptor pair transitions which occur due to the ternary QD intrinsic defects, while the fastest component has been explained by invoking the surface defects that provide local relaxation sites for the photoexcited electron-hole pairs [26,27]. It has also been demonstrated that the ternary QD photoluminescence lifetimes measured inside the photoluminescence (PL) band are emission-wavelength-dependent: the longer the wavelength, the longer the decay times [28].

Low toxicity and wavelength-dependent long photoluminescence decay times make the ternary QDs promising candidates for the time- and spectral-resolved bioimaging and sensing systems. It was shown that the fluorophore’s long lifetimes lead to a considerable suppression of the autofluorescence background and, consequently, to the overall sensitivity improvement [29]. Broad bandwidths and long lifetimes of ternary QDs are also beneficial for the applications with Förster resonance energy transfer (FRET), where QDs can be utilized as efficient energy donors [30,31,32,33]. In this case, the donor−acceptor pairs with acceptor absorption bands overlapping QD PL at different wavelengths and lifetimes result in different lifetimes of sensitized dye PL that allow for spectral and lifetime multiplexing [30,31,34]. The combined multiplexing strategy is crucial for creating the next generation bioanalytical sensing platforms and has been a subject of intensive research in the last few years [35,36].

The use of carrier particles, such as polymer microspheres (PMS), can significantly enhance the sensing platform’s versatility and capabilities. Microspheres are usually a few microns in size and can be encoded with organic dyes or quantum dots and functionalized with various biologically active molecules [37,38]. There are several advantages of using carrier matrices like PMS in imaging and sensing. Firstly, they increase the stability of the system by isolating the fluorophores from the environment and vice versa [39,40,41]. In the case of the PL-imaging, they lead to a strong increase in the signal intensity without the loss of spatial resolution by concentrating a large number of fluorophores in the volume of PMS. Finally, the combination of polymer microspheres and fluorophores with long lifetimes, such as ternary quantum dots, could make it possible to perform time-resolved barcoding and multitarget analysis using flow cytometry [42,43].

Despite active research, the field of PMS-based multiplex assays is still undeveloped. One of the main drawbacks is the utilization of toxic quantum dots or organic dyes in PMS based assays that do not provide the desired degree of multiplexing [44]. In the time-resolved analysis, nanoparticles doped with rare earth elements have also been used but they lack versatility due to the fixed lifetimes and PL band positions [45,46]. To address the abovementioned problems, here we have developed a new proof-of-concept sensing platform based on PMS encoded with the non-toxic AIS/ZnS QDs (PMS-AIS) and investigated its FRET with two different organic dyes. The designed system utilizes the time-resolved FRET from QDs (donors) to dyes (acceptors), which leads to the possibility of recording spectrally different bands of FRET-induced luminescence for each dye with different decay times. Given that the PL lifetimes of QDs increase with the wavelength, the sensitized luminescence lifetimes of the dyes depends on which part of the QD luminescence spectrum they absorb [31]. The system thereby enables to perform the potential multiplexing by wavelength and photoluminescence lifetimes of the dyes.

## 2. Materials and Methods

### 2.1. Materials

Indium(III) chloride tetrahydrate (InCl_3_·4H_2_O), silver nitrate (AgNO_3_), zinc(II) acetate dihydrate (Zn(CH_3_COO)_2_·2H_2_O), sodium sulfide nonahydrate (Na_2_S·9H_2_O), poly(allylamine hydrochloride) (PAH), poly(sodium 4-styrenesulfonate) (PSS), sodium chloride (NaCl), ammonium hydroxide solution (NH_4_OH), thioglycolic acid (TGA), isopropanol (i-PrOH), 4 nm polystyrene microspheres, 3,3′-diethylthiacarbocyanine iodide (Cy3) and 3,3′-diethylthiadicarbocyanine iodide (Cy5) were purchased from Sigma Aldrich and used without additional purification. All the solutions were prepared using deionized water as a solvent.

### 2.2. Synthesis of AIS/ZnS QDs

Hydrophilic synthesis of AgInS_2_/ZnS quantum dots was performed according to the previously described procedure [47,48]. This approach is based on the controllable reaction between sodium sulfide, silver (I) and indium (III) mercaptoacetate complexes. Briefly, AgNO_3_ was loaded in a three-necked round bottom flask filled with water under continuous stirring. After addition of TGA, the solution turned turbid. Then, the resulting solution turned yellow and transparent after adding NH_4_OH aqueous solution. Next, InCl_3_ in HNO_3_ 0.2 M aqueous solution was injected, that led to discoloration of the mixture because of the mercaptoacetate complexes formation. Then, Na_2_S solution was added and the solution became orange. After that the flask was heated up to 95 °C for 30 min with further addition of TGA, Zn(CH_3_COO)_2_ solution and NH_4_OH solutions for ZnS shell growth. Obtained mixture was one more time heated up to 95 °C for 30 min. At the end of the reaction, the flask was cooled down and the solution was rotary evaporated and further purified by size-selective precipitation using i-PrOH. The fracture of AIS/ZnS QDs of yellow color was redissolved in Millipore water for further use.

### 2.3. Preparation of AIS/ZnS-Doped Microspheres

One milliliter of a 0.5 M NaCl solution of a poly(allylamine hydrochloride) (PAH) polyelectrolyte with the concentration of 5 mg/mL was added to the precipitated polystyrene microspheres. The resulting dispersion was shaken for 10 min. To remove excess PAH polyelectrolyte, the solution was centrifuged for 30 s at 1500 rcf, the supernatant was removed and the precipitate was washed with distilled water. Then 1 mL of 0.5 M NaCl solution of a poly(sodium 4-styrenesulfonate) (PSS) polyelectrolyte with a concentration of 5 mg/mL was added to the coated spheres. The resulting dispersion was shaken for 10 min to remove an excess of PSS polyelectrolyte and then the solution was centrifuged for 30 s at 4000 rpm, the supernatant was removed and the precipitate was washed with distilled water. This polyelectrolyte coating and purification procedure was repeated until five PAH/PSS binary layers were formed. After the obtained ten-layer coating with polyelectrolytes, a positive PAH polyelectrolyte layer was formed on the surface by the similar method described above.

Then, 200 μL of a concentrated aqueous solution of AIS/ZnS QDs was added to the precipitated microspheres. The resulting dispersion was shaken overnight. To remove unreacted QDs, the solution was centrifuged for 30 s at 4000 rpm, the supernatant was removed and the precipitate was washed with distilled water. The resulting spheres with an outer layer of QDs are additionally covered with different numbers of PAH/PSS binary layers (1–5) by a similar method described for the previous layers (samples L1–L5).

Cy3 and Cy5 dyes were added to each sample with 1–5 PAH/PSS binary layers, samples were shaken for 5 min and washed 2 times with distilled water. The concentration of PMS and dyes slightly varied from sample to sample after washing procedure, which was determined from UV-Vis spectra. All PL spectra of the samples were corrected for the concentration.

### 2.4. Equipment

The UV-VIS absorption spectra were recorded using a UV-Probe 3600 spectrophotometer (Shimadzu, Kyoto, Japan). The steady-state PL spectra were obtained with Cary Eclipse spectrofluorometer (Agilent, Santa Clara, CA, USA). Time-resolved fluorescence spectroscopy measurements were performed using a time-correlated single photon counting (TCSPC) fluorescence microscope MicroTime 100 (PicoQuant, Inc. Inc., Berlin, Germany) equipped with a 405 nm pulsed laser LDH-P-C-405B (PicoQuant) with pulse duration of 20 ps. The signal was collected by a single photon photomultiplier tube detector; different detection wavelengths were selected with a holographic bandpass filter with the 10 nm bandwidth tunable in the spectral range of 430–780 nm. Laser frequency was adjusted using an additional signal generator SFG-71003 (Good Will Instek, Montclair, CA, USA). The fluorescent images were obtained with confocal laser scanning microscope LSM 710 (Carl Zeiss, Oberkochen, Germany) based on upright stand Axio Imager Z1 with objective EC Epiplan-Apochromat 50×/0.95. For excitation luminescence was used diode laser with 405 nm wavelength. PL signal was collected by the 32-channel spectral detector QUASAR.

## 3. Results and Discussion

### 3.1. Spectroscopy of AIS QDs and Cyanine Dyes

**Figure 1:**
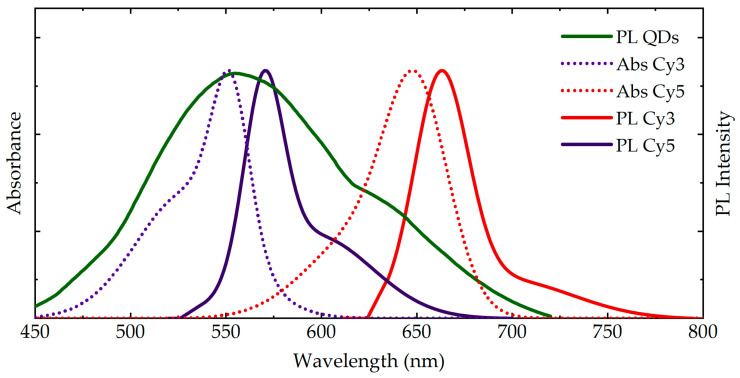
Absorption (dotted lines) and PL (solid lines) spectra of aqueous solutions of AgInS_2_/ZnS quantum dots (QDs) (green) and the cyanine dyes: Cy3 (purple) and Cy5 (red). PL excitation wavelengths: QDs—405 nm, Cy3—500 nm, Cy5—600 nm.

The normalized PL spectra of AgInS_2_/ZnS QDs (AIS QDs) and UV-Vis and PL spectra of Cy3 and Cy5 dyes are presented in Figure 1. The PL band maximum of AIS is 555 nm, full width at half maximum is 121 nm. The PL quantum yield is 38.6%. The quantum yield was estimated relatively against rhodamine 6G. The PL bands of Cy3 and Cy5 dyes have maxima at 570 nm and 663 nm, respectively. The donor (QD) PL and acceptors (Cy3 or Cy5) absorption bands overlap well, providing a possibility to FRET from QDs to the molecules. In our experiments, to facilitate the observation and analysis of the energy transfer from QD to dyes, PL of AgInS_2_/ZnS QDs was excited at 350 nm, where the absorption of dyes is negligible, while Cy3 and Cy5 were irradiated at 500 nm and 600 nm respectively since at these wavelengths the QDs do not absorb.

### 3.2. Confocal and Fluorescence Lifetime Imaging of PMS-AIS and PMS-AIS-Dyes

**Figure 2:**
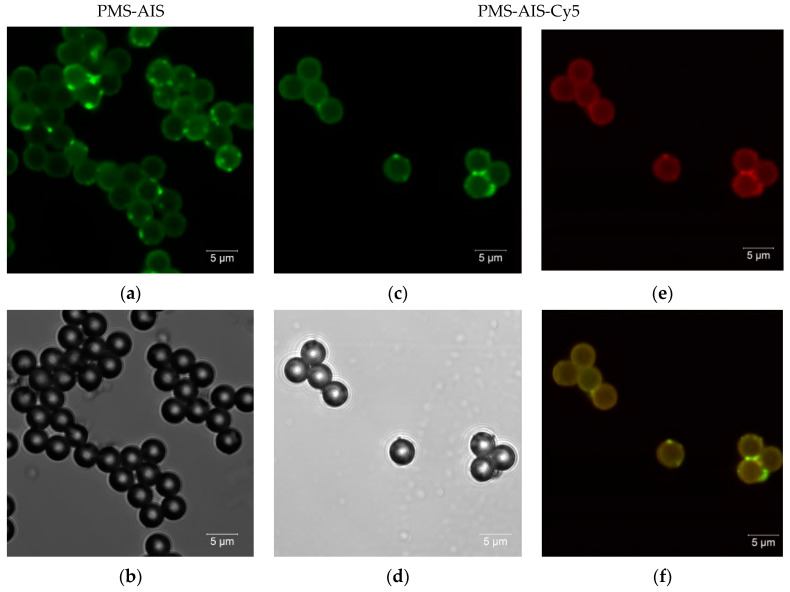
Confocal image of polystyrene microspheres with AIS QDs (PMS-AIS) and PMS-AIS-Cy5. PL (**a**) and transmission electron microscopy (TEM) (**b**) images of PMS-AIS; PL excitation at 405 nm, detection at 470–600 nm region. (**c**,**e**) correspond to the PL images of PMS-AIS-Cy5 excited by 405 nm radiation with PL detection at 470–600 nm and 650–750 nm, respectively and their superposition (**f**). (**d**) TEM images of PMS-AIS-Cy5.

As a typical example, confocal PL and transmission images of microspheres with QDs on the surface (PMS-AIS) as well as with QDs and Cy5 dye (PMS-AIS-Cy5) are shown in Figure 2. The PL image of PMS-AIS was registered with an excitation wavelength of 405 nm and emission wavelength region of 450–600 nm (Figure 2a). QDs cover the PMS surface quite evenly but in some regions bright spots are observed, probably associated with an increased local concentration of the QDs. PL images of the complexes PMS-AIS-Cy5 were excited by the 405 nm radiation and registered at 450–580 nm for PL of QDs (Figure 2c) and 660–730 nm for FRET-induced sensitized PL of Cy5 (Figure 2e) by the 32-channel spectral detector. Figure 2f represents the superposition of the QD and Cy5 PL. The bright spots of Cy5 sensitized PL correspond to QD bright PL areas as FRET occurs more intensively in these regions.

### 3.3. UV-Vis Spectroscopy of PMS-AIS and PMS-AIS-Dyes

**Figure 3:**
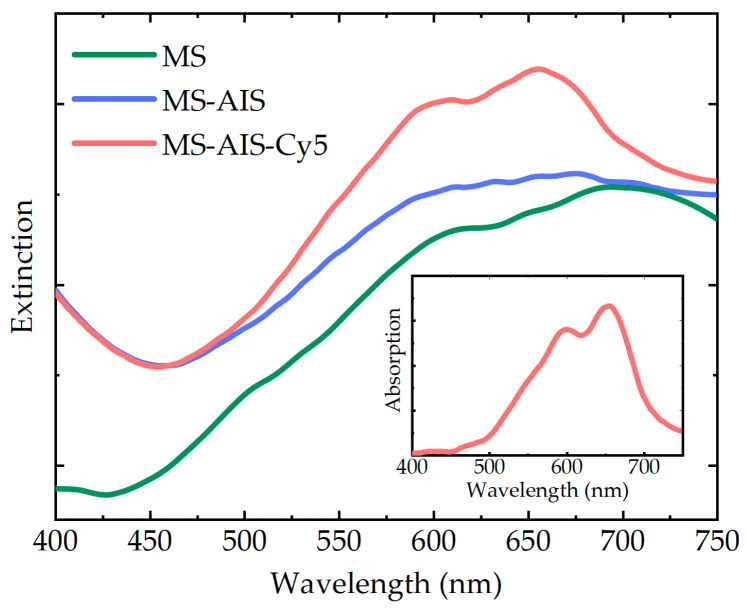
Extinction spectra of MS, PMS-AIS and PMS-AIS-Cy5. Inset: Cy5 absorption is revealed in PMS-AIS-Cy5 after the subtraction of the PMS-AIS background.

Microspheres have strong scattering due to the relatively large particle size, which is reflected in their extinction spectra as an appearance of a very high background (Figure 3). Absorption spectra of dyes comprised of PMS-AIS-dye complexes were obtained by subtraction of the background. Figure 3 demonstrates, as an example, the absorption spectra of Cy5 dye in the PMS-AIS-Cy5 complexes, which has two absorption bands: 595 nm and 647 nm. The peak at 647 nm is also observed in the absorption spectrum of Cy5 in a free state and corresponds to the monomeric form. The peak at 595 nm corresponds to the Cy5 dimer [28] and its appearance indicates a partial aggregation of the dye upon adsorption on PMS-AIS.

### 3.4. PL Spectra of PMS-AIS and PMS-AIS-Dyes Excited at 350 nm

**Figure 4:**
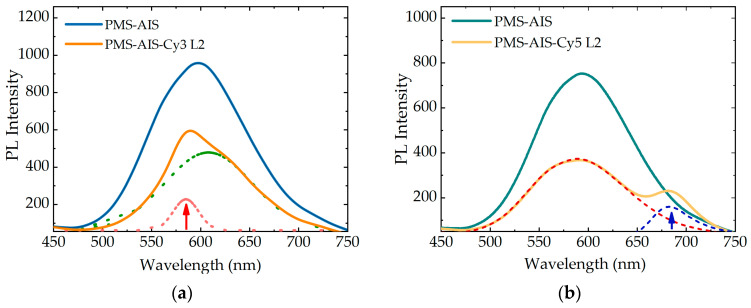

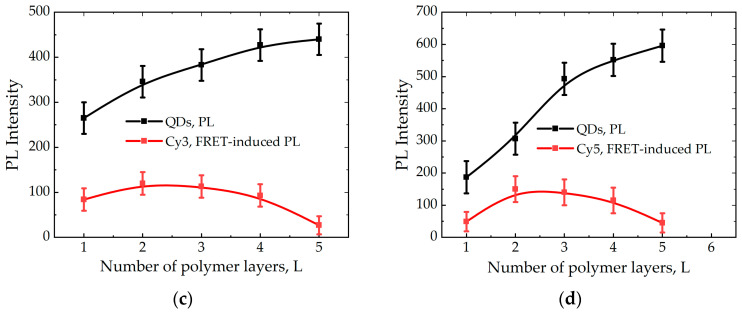
(**a**) Photoluminescence (PL) spectra of PMS-AIS, PMS-AIS-Cy3 L2 and deconvolution of PMS-AIS-Cy3 L2 by AIS and Cy3 PL band. (**c**) Dependence of QD PL and Cy3 FRET-induced PL on number of polymer layers L. (**b**) PL spectra of PMS-AIS, PMS-AIS-Cy5 L2 and deconvolution of PMS-AIS-Cy5 L2 by AIS and Cy5 PL band. (**d**) Dependence of QD PL and Cy5 FRET-induced PL on number of polymer layers L.

Figure 4 represents PL spectra of PMS-AIS and PMS-AIS-dye excited at 350 nm, where QDs absorb the light, while the dye does not have absorption and, therefore, cannot be directly excited. As a result of FRET, the QD PL is quenched and the dye sensitized PL appears. For clarity, the PL spectra of PMS-AIS-dyes with two double spacer layers of polyelectrolytes (L2) are deconvoluted by gaussian AIS and Cy3 PL bands. To address the distance dependence of the photoexcitation energy transfer from QDs to dyes, we studied the quenching of the QD luminescence and the change in the intensity of the sensitized PL of dyes as a function of the number of polyelectrolyte layers. The thickness of one double PAH/PSS layer is approximately 1 nm [49], consequently, 5 layers are approximately 5 nm. Calculated FRET radii of the QD-Cy3 and QD-Cy5 pairs are 5.39 and 6.67 nm, respectively. We found that the degree of PL quenching increases monotonically with a decrease in the number of layers and QD PL is quenched most intensively for PMS-AIS-dyes without any and with one double layer of polyelectrolytes (1L). It is reasonable, since, in addition to FRET, charge carrier transfer, usually responsible for the most effective QD PL quenching process, prevails at 1–2 nm distance between a QD and a dye molecule. The sensitized PL of the dyes also drops with the number of layers since the efficiency of FRET decreases with distance. However, for the PMS-AIS-dyes, the sensitized PL intensity possesses a noticeable maximum at L2. This is most likely due to the appearance of an additional nonradiative energy relaxation channel when the distance between QDs and dye molecules becomes close enough for electron transfer from QDs to dyes. Thus, the samples with 2–3 polymer layers have optimal conditions for FRET, because the distance between the QDs and the dyes is less than the Förster radius and the FRET efficiency is high. Further, for samples L4 and L5, the FRET efficiency decreases.

### 3.5. PL Spectra of PMS-AIS and PMS-AIS-Dyes Excited at 520 and 600 nm

**Figure 5:**
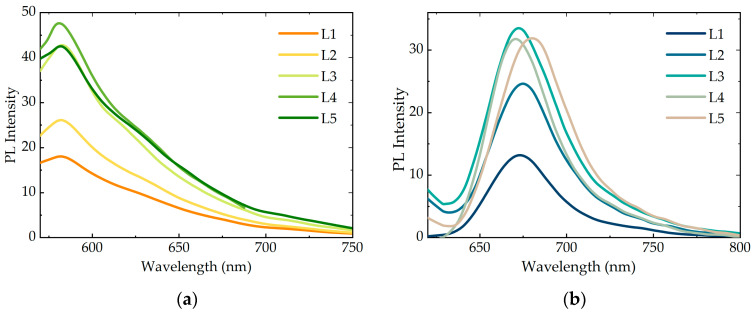
PL spectra of (**a**) PMS-AIS-Cy3 and (**b**) PMS-AIS-Cy5 with 1-5 double layers of polyelectrolytes. Excitation wavelength is 520 and 600 nm, respectively.

Since in the present system, there is a potential competition between the sensitized PL of the dyes and quenching of the luminescence of dyes due to the electron transfer, which have different distance dependences, we studied the effect of the polyelectrolyte spacer layer thickness on the intensity of the dye intrinsic PL in the PMS-AIS-dye. The intrinsic PL spectra of Cy3 and Cy5 excited at 520 and 600 nm, respectively, where QDs do not absorb light, are shown in Figure 5. Spectra were corrected to dye concentration. A noticeable quenching of the dye PL is observed for L1 and L2, being the most pronounced for L1, which confirms the assumption of additional nonradiative channel formation in the vicinity of the QDs. The luminescence of the samples L3–L5 is approximately at the same level.

### 3.6. Time-Resolved PL Measurements of PMS-AIS and PMS-AIS-Dyes

For the implementation of TR-FRET, it is important that the luminescence lifetime of AIS QDs depends on the wavelength of the detected radiation. The AIS QDs PL decay curves measured at different detection wavelengths were well fitted by two exponents:

(1) $$I\left(t\right)=A_{1}e^{-t/\tau_{1}}+A_{2}e^{-t/\tau_{2}}.$$

The average PL lifetimes was calculated by the following equation:

(2) $$<\tau >=\frac{A_{1}\tau_{1}^{2}+A_{2}\tau_{2}^{2}}{A_{1}\tau_{1}+A_{2}\tau_{2}},$$

where $A_{1}$ and $A_{2}$ are the amplitudes and $\tau_{1}$ and $\tau_{2}$ are the decay times of the first and second exponents, respectively. The values of these parameters are shown in Table 1.

**Figure 6:**
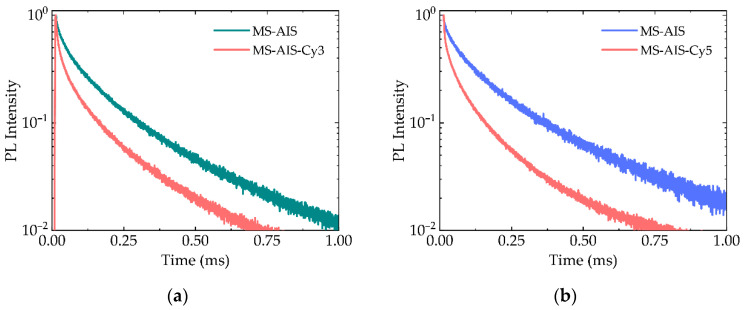
PL decay curves of (**a**) PMS-AIS (green) and PMS-AIS-Cy3 L2 (red) registered at the 580 nm and (**b**) PMS-AIS (blue) and PMS-AIS-Cy5 L2 (red) recorded at the 665 nm.

The absorption bands of Cy3 and Cy5 overlap different spectral regions of the AIS QD PL band having different lifetimes. Due to FRET, the PL lifetime decreases for QDs and increases for dyes (Figure 6).

Intrinsic PL lifetimes of Cy3 and Cy5 are approximately 0.6 and 0.5 ns, respectively. In complexes with QDs the PL lifetime of Cy3 and Cy5 increased to 20 and 30 ns at the maxima of their PL bands 580 and 665 nm, respectively (Table 1). The AIS QDs have PL lifetimes increasing with PL excitation wavelengths, as was shown previously [50]. Since Cy3 has an absorption band overlapping with the QD PL band at lower wavelengths with shorter lifetimes than Cy5, the lifetime of sensitized PL of Cy3 is also smaller, which can be used in time-resolved multiplexing.

**Figure 7:**
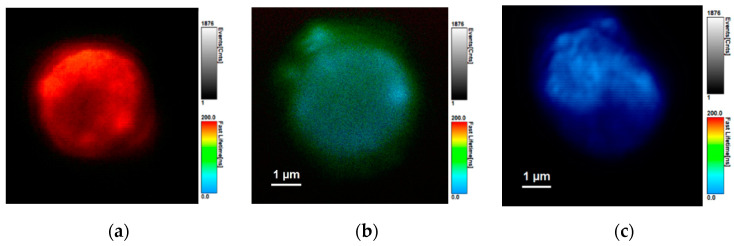
Fluorescence lifetime images of the microspheres doped by QDs and QDs with Cy3 or Cy5 dyes measured at maxima of QD PL and the FRET-induced dyes PL. (**a**) PMS-AIS ($\lambda_{PL}$ = 665 nm, <τ> = 245 ns), (**b**) PMS-AIS-Cy5 L2 ($\lambda_{PL}$ = 665 nm, <τ> = 160 ns) and (**c**) PMS-AIS-Cy3 L2 ($\lambda_{PL}$ = 580 nm, <τ> = 80 ns). PL wavelengths (*λ_PL_*) and corresponding average PL decay times <*τ*> are shown. The color scales of PL lifetimes are shown.

Figure 7 presents the FLIM of PMS-AIS, PMS-AIS-Cy3 and PMS-AIS-Cy5 measured at maxima of corresponding PL bands using the 10 nm bandpass filters, demonstrating the significant change in the lifetimes at the wavelength of sensitized dye PL. These images illustrate the ability of the developed PMS-AIS-dyes system for multiplexed sensing with conventional bandpass spectral techniques and time selection techniques.

## 4. Conclusions

In this paper, we have developed a multiplex assay model that is based on polymer microspheres encoded with AIS/ZnS QDs and investigated their interaction with two different cyanine dyes emitting in different spectral regions. Our studies have shown that the QD PL quenching steadily increases with the decrease in the number of polyelectrolyte layers between the QDs and the dyes. As a result, the QD PL quenches the most for PMS-AIS-dyes with no or just one double layer of polyelectrolytes. However, the sensitized dye PL intensity demonstrates a clear maximum in the case of two double layers in PMS-AIS-dyes, which has been attributed to the presence of an additional nonradiative energy relaxation channel when the distance between QDs and dye molecules. In addition, direct excitation of the dyes has demonstrated a visible quenching for the samples with one and two double layers of coating confirming the presence of an additional nonradiative channel in the vicinity of the QDs. Time-resolved PL measurements of PMS-AIS and PMS-AIS-dyes systems have shown that the PL lifetime decreases for QDs and increases for dyes because of FRET. PL lifetimes of AIS QDs also increases with the PL excitation wavelength and Cy3 dye’s absorption overlaps with the QD PL band at lower wavelengths with shorter lifetimes than those for Cy5 dye. The study clearly demonstrates the potential of the proposed model to record the spectrally separated bands of FRET-induced luminescence for dyes with different decay times. Therefore, these PMS-based systems can be used to achieve multiplexing by wavelength and photoluminescence lifetimes of the dyes. We also believe that PMS encoded with AIS/ZnS QDs have a potential for design of highly selective and sensitive sensors for multiplex analysis to detect cell lysates and body fluids’ representative biomarkers.

## Figures and Tables

**Table 1:** PL lifetimes of PMS-AIS, PMS-AIS-Cy3 and PMS-AIS-Cy5 measured at 580 and 665 nm.

Sample Name	$\tau_{1}\;(\mathrm{ns})$	$\tau_{2}\;(\mathrm{ns})$	$\tau_{3}\;(\mathrm{ns})$	<τ> (ns)
PMS-AIS (580 nm)	460 ± 5	130 ± 5	—	130 ± 5
PMS-AIS-Cy3 (580 nm)	440 ± 5	120 ± 5	20 ± 5	80 ± 5
PMS-AIS (665 nm)	745 ± 5	225 ± 5	—	245 ± 5
PMS-AIS-Cy5 (665 nm)	705 ± 5	210 ± 5	30 ± 5	160 ± 5

## Data Availability

The study does not report data which present the ethical, legal or privacy issues. The data can be shared.

## References

[1] Howes P.D., Chandrawati R., Stevens M.M. (2014). Colloidal nanoparticles as advanced biological sensors. Science.

[2] Volkov Y. (2015). Quantum dots in nanomedicine: Recent trends, advances and unresolved issues. Biochem. Biophys. Res. Commun..

[3] Ramasamy P., Kim N., Kang Y.-S., Ramirez O., Lee J.-S. (2017). Tunable, Bright, and Narrow-Band Luminescence from Colloidal Indium Phosphide Quantum Dots. Chem. Mater..

[4] Willard D.M., Carillo L.L., Jung J., Van Orden A. (2001). CdSe−ZnS Quantum Dots as Resonance Energy Transfer Donors in a Model Protein−Protein Binding Assay. Nano Lett..

[5] Hyldahl M.G., Bailey S.T., Wittmershaus B.P. (2009). Photo-stability and performance of CdSe/ZnS quantum dots in luminescent solar concentrators. Sol. Energy.

[6] Hao J., Zhou J., Zhang C. (2013). A tri-n-octylphosphine-assisted successive ionic layer adsorption and reaction method to synthesize multilayered core–shell CdSe–ZnS quantum dots with extremely high quantum yield. Chem. Commun..

[7] Zhang H., Hu N., Zeng Z., Lin Q., Zhang F., Tang A., Jia Y., Li L.S., Shen H., Teng F. (2019). High-Efficiency Green InP Quantum Dot-Based Electroluminescent Device Comprising Thick-Shell Quantum Dots. Adv. Opt. Mater..

[8] Frecker T., Bailey D., Arzeta-Ferrer X., McBride J., Rosenthal S.J. (2015). Review—Quantum Dots and Their Application in Lighting, Displays, and Biology. ECS J. Solid State Sci. Technol..

[9] Resch-Genger U., Grabolle M., Cavaliere-Jaricot S., Nitschke R., Nann T. (2008). Quantum dots versus organic dyes as fluorescent labels. Nat. Methods.

[10] Schliwa A., Winkelnkemper M., Bimberg D. (2007). Impact of size, shape, and composition on piezoelectric effects and electronic properties of In (Ga) As/Ga As quantum dots. Phys. Rev. B.

[11] Ngo C.Y., Yoon S.F., Fan W.J., Chua S.J. (2006). Effects of size and shape on electronic states of quantum dots. Phys. Rev. B.

[12] Kershaw S.V., Susha A.S., Rogach A.L. (2013). Narrow bandgap colloidal metal chalcogenide quantum dots: Synthetic methods, heterostructures, assemblies, electronic and infrared optical properties. Chem. Soc. Rev..

[13] Girma W.M., Fahmi M.Z., Permadi A., Abate M.A., Chang J.-Y. (2017). Synthetic strategies and biomedical applications of I–III–VI ternary quantum dots. J. Mater. Chem. B.

[14] Long Z., Zhang W., Tian J., Chen G., Liu Y., Liu R. (2020). Recent research on the luminous mechanism, synthetic strategies, and applications of CuInS2 quantum dots. Inorg. Chem. Front..

[15] Ghasemi Y., Peymani P., Afifi S. (2009). Quantum dot: Magic nanoparticle for imaging, detection and targeting. Acta Biomed..

[16] Hines M.A., Scholes G.D. (2003). Colloidal PbS nanocrystals with size-tunable near-infrared emission: Observation of post-synthesis self-narrowing of the particle size distribution. Adv. Mater..

[17] Li J.J., Wang Y.A., Guo W., Keay J.C., Mishima T.D., Johnson M.B., Peng X. (2003). Large-Scale Synthesis of Nearly Monodisperse CdSe/CdS Core/Shell Nanocrystals Using Air-Stable Reagents via Successive Ion Layer Adsorption and Reaction. J. Am. Chem. Soc..

[18] Tsoi K.M., Dai Q., Alman B.A., Chan W.C.W. (2013). Are Quantum Dots Toxic? Exploring the Discrepancy Between Cell Culture and Animal Studies. Acc. Chem. Res..

[19] Oh E., Liu R., Nel A., Gemill K.B., Bilal M., Cohen Y., Medintz I.L. (2016). Meta-analysis of cellular toxicity for cadmium-containing quantum dots. Nat. Nanotechnol..

[20] Kurshanov D., Gromova Y., Cherevkov S., Ushakova E., Kormilina T., Dubavik A., Fedorov A., Baranov A. (2018). Non-Toxic Ternary Quantum Dots AgInS2 and AgInS2/ZnS: Synthesis and Optical Properties. Opt. Spectrosc..

[21] Mao B., Chuang C.-H., Lu F., Sang L., Zhu J., Burda C. (2013). Study of the Partial Ag-to-Zn Cation Exchange in AgInS2/ZnS Nanocrystals. J. Phys. Chem. C.

[22] Dai M., Ogawa S., Kameyama T., Okazaki K., Kudo A., Kuwabata S., Tsuboi Y., Torimoto T. (2012). Tunable photoluminescence from the visible to near-infrared wavelength region of non-stoichiometric AgInS2 nanoparticles. J. Mater. Chem..

[23] Chevallier T., Le Blevennec G., Chandezon F. (2016). Photoluminescence properties of AgInS2–ZnS nanocrystals: The critical role of the surface. Nanoscale.

[24] Zhong H., Zhou Y., Ye M., He Y., Ye J., He C., Yang C., Li Y. (2008). Controlled Synthesis and Optical Properties of Colloidal Ternary Chalcogenide CuInS2 Nanocrystals. Chem. Mater..

[25] Sun J., Ikezawa M., Wang X., Jing P., Li H., Zhao J., Masumoto Y. (2015). Photocarrier recombination dynamics in ternary chalcogenide CuInS2 quantum dots. Phys. Chem. Chem. Phys..

[26] Cai C., Zhai L., Ma Y., Zou C., Zhang L., Yang Y., Huang S. (2017). Synthesis of AgInS2 quantum dots with tunable photoluminescence for sensitized solar cells. J. Power Sources.

[27] Xiang W., Xie C., Wang J., Zhong J., Liang X., Yang H., Luo L., Chen Z. (2014). Studies on highly luminescent AgInS2 and Ag–Zn–In–S quantum dots. J. Alloys Compd..

[28] Ogawa T., Kuzuya T., Hamanaka Y., Sumiyama K. (2010). Synthesis of Ag–In binary sulfide nanoparticles—structural tuning and their photoluminescence properties. J. Mater. Chem..

[29] Wegner K.D., Lanh P.T., Jennings T., Oh E., Jain V., Fairclough S.M., Smith J.M., Giovanelli E., Lequeux N., Pons T. (2013). Influence of Luminescence Quantum Yield, Surface Coating, and Functionalization of Quantum Dots on the Sensitivity of Time-Resolved FRET Bioassays. ACS Appl. Mater. Interfaces.

[30] Miropoltsev M., Kuznetsova V., Tkach A., Cherevkov S., Sokolova A., Osipova V., Gromova Y., Baranov M., Fedorov A., Gun’ko Y. (2020). FRET-Based Analysis of AgInS_2_/ZnAgInS/ZnS Quantum Dot Recombination Dynamics. Nanomaterials.

[31] Kuznetsova V., Tkach A., Cherevkov S., Sokolova A., Gromova Y., Osipova V., Baranov M., Ugolkov V., Fedorov A., Baranov A. (2020). Spectral-Time Multiplexing in FRET Complexes of AgInS_2_/ZnS Quantum Dot and Organic Dyes. Nanomaterials.

[32] Evstigneev R., Parfenov P., Dubavik A., Cherevkov S., Fedorov A., Martynenko I., Resch-Genger U., Ushakova E., Baranov A. (2019). Time-resolved FRET in AgInS2/ZnS-CdSe/ZnS quantum dot systems. Nanotechnology.

[33] Martynenko I., Kuznetsova V., Orlova A., Kanaev P., Gromova Y., Maslov V., Baranov A., Fedorov A. ZnSe/ZnS quantum dots—Photosensitizer complexes: Optical properties and cancer cell photodynamic destruction effect. Proceedings of the SPIE—The International Society for Optical Engineering, SPIE Photonics Europe.

[34] Martynenko I.V., Baimuratov A.S., Weigert F., Soares J.X., Dhamo L., Nickl P., Doerfel I., Pauli J., Rukhlenko I.D., Baranov A.V. (2019). Photoluminescence of Ag-In-S/ZnS quantum dots: Excitation energy dependence and low-energy electronic structure. Nano Res..

[35] Petryayeva E., Algar W.R., Medintz I.L. (2013). Quantum Dots in Bioanalysis: A Review of Applications Across Various Platforms for Fluorescence Spectroscopy and Imaging. Appl. Spectrosc..

[36] Snee P.T. (2020). Semiconductor quantum dot FRET: Untangling energy transfer mechanisms in bioanalytical assays. TrAC Trends Anal. Chem..

[37] Zhang Y., Dong C., Su L., Wang H., Gong X., Wang H., Liu J., Chang J. (2016). Multifunctional Microspheres Encoded with Upconverting Nanocrystals and Magnetic Nanoparticles for Rapid Separation and Immunoassays. ACS Appl. Mater. Interfaces.

[38] Han M., Gao X., Su J.Z., Nie S. (2001). Quantum-dot-tagged microbeads for multiplexed optical coding of biomolecules. Nat. Biotechnol..

[39] Buranda T., Huang J., Ramarao G.V., Ista L.K., Larson R.S., Ward T.L., Sklar L.A., Lopez G.P. (2003). Biomimetic Molecular Assemblies on Glass and Mesoporous Silica Microbeads for Biotechnology. Langmuir.

[40] Sonawane S.L., Asha S.K. (2016). Fluorescent Polystyrene Microbeads as Invisible Security Ink and Optical Vapor Sensor for 4-Nitrotoluene. ACS Appl. Mater. Interfaces.

[41] Gao X., Nie S. (2005). Quantum dot-encoded beads. NanoBiotechnology Protocols.

[42] Kage D., Hoffmann K., Nifontova G., Krivenkov V., Sukhanova A., Nabiev I., Resch-Genger U. (2020). Tempo-spectral multiplexing in flow cytometry with lifetime detection using QD-encoded polymer beads. Sci. Rep..

[43] Kage D., Hoffmann K., Wittkamp M., Ameskamp J., Göhde W., Resch-Genger U. (2018). Luminescence lifetime encoding in time-domain flow cytometry. Sci. Rep..

[44] Bilan R., Nabiev I., Sukhanova A. (2016). Quantum dot-based nanotools for bioimaging, diagnostics, and drug delivery. ChemBioChem.

[45] Ma Q., Wang J., Li Z., Lv X., Liang L., Yuan Q. (2019). Recent Progress in Time-Resolved Biosensing and Bioimaging Based on Lanthanide-Doped Nanoparticles. Small.

[46] Wolska E., Kaszewski J., Kiełbik P., Grzyb J., Godlewski M.M., Godlewski M. (2014). Rare earth activated ZnO nanoparticles as biomarkers. Opt. Mater..

[47] Raevskaya A., Lesnyak V., Haubold D., Dzhagan V., Stroyuk O., Gaponik N., Zahn D.R.T., Eychmüller A. (2017). A Fine Size Selection of Brightly Luminescent Water-Soluble Ag–In–S and Ag–In–S/ZnS Quantum Dots. J. Phys. Chem. C.

[48] Gromova Y., Sokolova A., Kurshanov D., Korsakov I., Osipova V., Cherevkov S., Dubavik A., Maslov V., Perova T., Gun’ko Y. (2019). Investigation of AgInS2/ZnS Quantum Dots by Magnetic Circular Dichroism Spectroscopy. Materials.

[49] Michel M., Toniazzo V., Ruch D., Vincent B. (2012). Deposition Mechanisms in Layer-by-Layer or Step-by-Step Deposition Methods: From Elastic and Impermeable Films to Soft Membranes with Ion Exchange Properties. ISRN Mater. Sci..

[50] Hamanaka Y., Ozawa K., Kuzuya T. (2014). Enhancement of Donor–Acceptor Pair Emissions in Colloidal AgInS2 Quantum Dots with High Concentrations of Defects. J. Phys. Chem. C.

